# The Production of Braille Books

**Published:** 1914-03-28

**Authors:** C. Arthur Pearson

**Affiliations:** National Institute for the Blind. Hon. Treasurer


*To the Editor of* The Hospital.

### The Production of Braille Books.

*To the Editor of* The Hospital.

Sir,—I wonder if you will allow me a little space in which to talk of a rather important point with regard to the production of Braille books. There are many kindly sighted people in this country who make these books by hand, and this is also done at various institutions by blind people, particularly at the National Lending Library, so ably managed by Miss Austin.

But the work is necessarily extremely slow, and the entire production of such books does not supply a fraction of the demand for Braille reading matter. I should like by your courtesy to suggest that in future Braille books thus produced should be of a special nature, and not, as is usually the case at present, books of general interest which can so much better be made in large quantities by machinery. There are many people who cannot see to read, but have some special pursuit or some special hobby with regard to which they require books that, though of great interest to them, are not of sufficient general interest to warrant their production in quantities. I want to establish a department here, the object of which will be the carrying out of this idea, provided that a sufficient demand and means of supply exist. I shall be very glad if people who are dependent upon Braille for reading will communicate with me, mentioning any particular book of a really special nature which they would like, and if kindly folk who are prepared to make such books will also let me hear from them. The rest will be easy, and I feel sure that great advantages will result.

I was led to this idea by being told by our Chairman, Dr. Ranger (who possesses, I believe, a unique Braille library), that for many years past two ladies have devoted much of their leisure time to making him Braille books on special subjects.

I hope this letter may perhaps have the result of increasing the number of people who engage in the kindly task of making Braille books by hand. The work is quite simple and quite easily learnt, and I am sure that much time which is now spent on comparatively useless occupations could be with great advantage employed for this.—Yours faithfully, C. Arthur Pearson,
National Institute for the Blind. Hon. Treasurer.

